# Stage‐Dependent Changes in Kynurenine Pathway Enzyme Expression Suggest Immune‐Related Involvement in Bladder Cancer Progression

**DOI:** 10.1111/iju.70491

**Published:** 2026-05-07

**Authors:** Douglas Edgard Lemes, Aline Áurea de Souza Santos, Jéssica Lopes de Oliveira, Henrique Roman‐Ramos, Ana Carolina Ramos Moreno, Cleber Pinto Camacho, José Pontes‐Junior, Humberto Dellê

**Affiliations:** ^1^ Molecular Innovation and Biotechnology Laboratory, Postgraduate Program in Medicine Universidade Nove de Julho (Uninove) São Paulo SP Brazil; ^2^ Vaccine Development Laboratory, Department of Microbiology Institute of Biomedical Sciences, University of São Paulo São Paulo SP Brazil; ^3^ Vaccine Development Laboratory Butantan Institute São Paulo SP Brazil; ^4^ Thyroid Diseases Center, Laboratory of Molecular and Translational Endocrinology, Division of Endocrinology, Department of Medicine, Escola Paulista de Medicina Universidade Federal de São Paulo São Paulo SP Brazil; ^5^ Urology Department, LIM/55 ‐ Laboratory of urology Hospital das Clínicas da Faculdade de Medicina da Universidade de São Paulo, Universidade de São Paulo, Instituto do Câncer do Estado de São Paulo São Paulo SP Brazil

**Keywords:** bladder cancer, immuno escape, indoleamine 2,3‐dioxygenase‐1, kynurenine, tryptophan

## Abstract

L‐tryptophan (Trp) metabolism through the L‐kynurenine (Kyn) pathway contributes to immune escape in neoplasms, including bladder cancer (BC). Indoleamine 2,3‐dioxygenase‐1 (IDO1) is a key enzyme, and its catabolites—Kyn, 3‐hydroxykynurenine (3HK), and 3‐hydroxyanthranilate (3HAA)—depend on other enzymes and play a crucial role in immunomodulation.

**Objectives:**

We investigated the association between Kyn pathway enzyme expression and BC prognostic factors.

**Methods:**

Data from 165 bc patients in the GEO DataSets were analyzed, comparing staging, tumor grade, progression, and recurrence with the expression of IDO1, arylformamidase (AFMID), kynurenine–oxoglutarate transaminase (KAT), kynureninase (KYNU), kynurenine 3‐monooxygenase (KMO), 3‐hydroxyanthranilate 3,4‐dioxygenase (HAAO), and aminocarboxymuconate‐semialdehyde decarboxylase (ACMSD). Predictive genes were further evaluated in RT4 (low‐grade, non‐invasive) and T24 (high‐grade, invasive) BC cells treated with IFN‐gamma and the IDO1 inhibitor INCB024360. Trp catabolites were quantified in the supernatant.

**Results:**

Enzyme expression varied widely, especially for IDO1, AFMID, KAT, KMO, and KYNU. IDO1 correlated positively with KMO and negatively with KAT. High IDO1 and KMO levels were linked to advanced‐stage disease, while KAT and KYNU were associated with earlier stages and lower tumor grade. RT4 and T24 cells showed distinct basal enzyme expression, with T24 cells being more responsive to IFN‐gamma, increasing IDO1 and KYNU while decreasing KAT and KMO. These effects were inhibited by INCB024360. High Trp consumption increased Kyn and 3HAA but not 3HK.

**Conclusions:**

Kyn pathway enzyme expression varies with disease progression and may indicate immune activity by influencing tumor microenvironment catabolites. BC cell sensitivity to immune stimuli differs, potentially shaping distinct immune escape mechanisms.

## 1 Introduction

Bladder cancer (BC) is the fourth most common malignancy in men, with a male‐to‐female ratio of 3:1 [1]. Non‐muscle‐invasive bladder cancer (NMIBC) constitutes 75% of the estimated 83 190 new cases in the United States for 2024. The global 5‐year prevalence in 2020 exceeded 1.5 million [1]. Muscle‐invasive bladder cancer (MIBC), the most advanced stage, carries a high metastasis risk. While current treatments are effective, new alternatives, including immunotherapy, are under investigation [2].

L‐tryptophan (Trp) catabolism via indoleamine 2,3‐dioxygenase‐1 (IDO1) contributes to immune evasion in tumors by promoting peripheral tolerance. IDO1's role in protecting the semi‐allogeneic embryo from maternal immune rejection was first described by Munn et al. [3], Trp depletion leads to L‐kynurenine (Kyn) formation, inducing T lymphocyte anergy and apoptosis, thereby ensuring local immune tolerance [4].

Clinical trials combining PD‐1/PD‐L1 blockade with IDO1 inhibition (Epacadostat, INCB024360) have produced mixed results. In advanced melanoma, Epacadostat did not improve outcomes when added to PD‐1/PD‐L1 therapy in a phase III trial [5]. In urothelial carcinoma, the phase III ECHO‐303/KEYNOTE‐698 study reported acceptable tolerability and antitumor activity, although final results are still pending [6].

**FIGURE 1:**
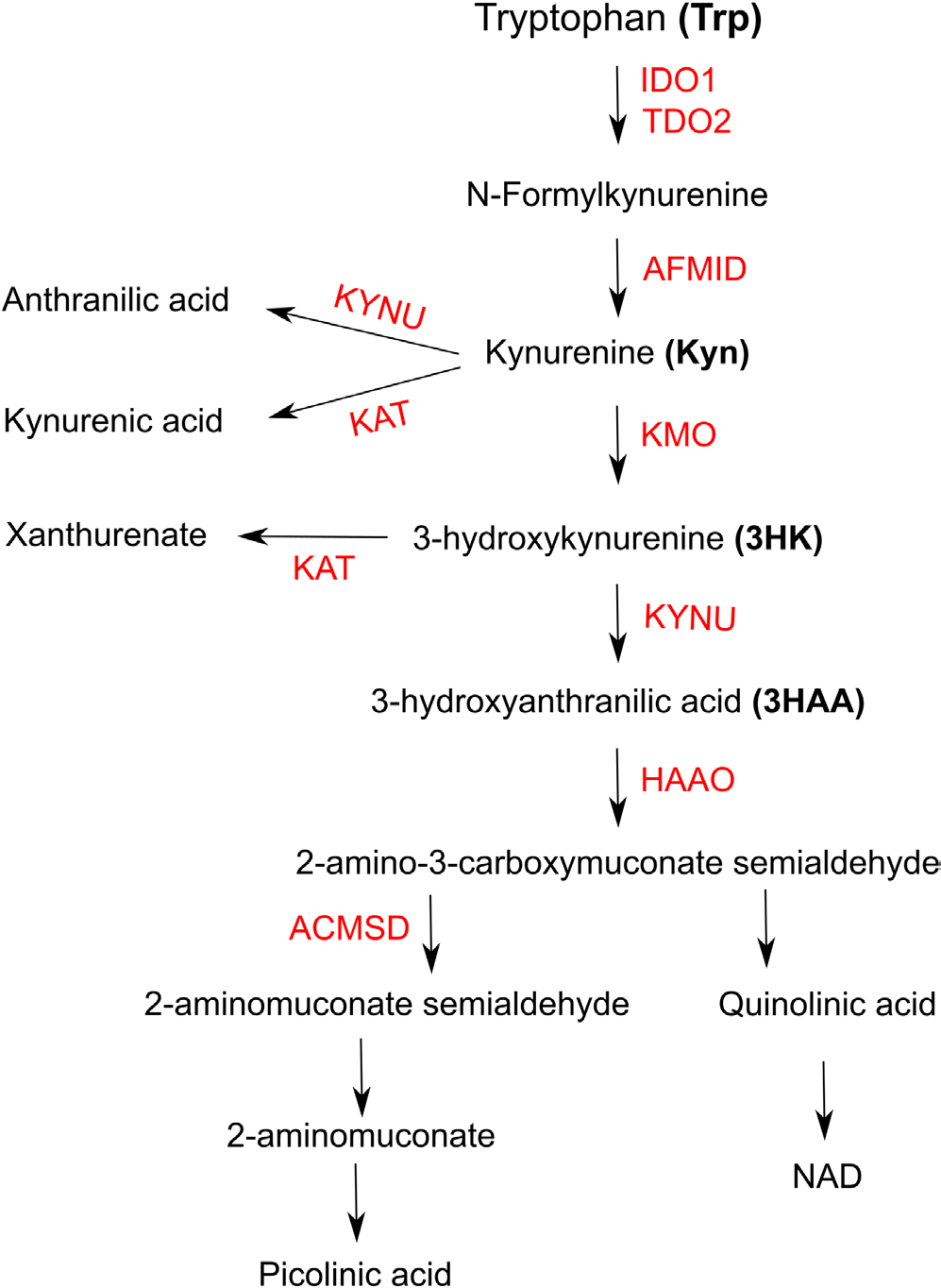
Catabolism of Trp through Kyn pathway. The enzymes are represented in red: Indoleamine 2,3‐dioxygenase‐1 (IDO1), Tryptophan 2,3‐dioxygenase‐2 (TDO2), arylformamidase (AFMID), kynurenine—oxoglutarate transaminase (KAT), kynureninase (KYNU), kynurenine 3‐monooxygenase (KMO), 3‐hydroxyanthranilate 3,4‐dioxygenase (HAAO), and aminocarboxymuconate‐semialdehyde decarboxylase (ACMSD).

The partial success of IDO1 inhibitor therapy may be due to the difficulty in assessing the Kyn pathway status in tumors. IDO1 expression via immunohistochemistry/fluorescence and RT‐qPCR does not guarantee functional enzymatic activity, as it depends on reducing agents and is vulnerable to local inhibitors [7]. This assessment is challenging since measuring IDO1 activity in the tumor microenvironment is complex. Many studies use the Kyn/Trp ratio in plasma, but this approach's effectiveness is debated, as plasma Trp depletion does not always correspond to increased Kyn [8]. Additionally, Kyn synthesis might occur in the tumor, but subsequent catabolites depend on other enzymes in the Kyn pathway (Figure 1). The combination of Kyn catabolites in the microenvironment may influence the tumor's immune status. Terness and colleagues showed that IDO1's effect on T lymphocytes and immune cells relies on both Trp depletion and the local production of Kyn and its derivatives, including 3‐hydroxykynurenine (3HK) and 3‐hydroxyanthranilic acid (3HAA), which induce T cell anergy and death when combined [9].

We hypothesize that the Kyn pathway may vary in BC, as occurs in other neoplasms [10]. Analyzing the expression of different enzymes in the Kyn pathway can provide insights into its status, potentially serving as a modifiable risk factor and a crucial element in therapeutic decision‐making.

## 2 Methods

### 2.1 Expression Analysis in the GEO DataSets Database

To analyze the expression of enzymes involved in Trp catabolism in human BC specimens, the Gene Expression Omnibus (GEO) database (http://www.ncbi.nlm.nih.gov/geo/) was used [11]. In addition to transcriptome data, information on tumor grade, stage, and progression was required. The GSE13507 dataset was selected, which includes data from 165 bc patients [12, 13]. GEO2R, an R‐based web tool designed for analyzing GEO datasets, was utilized to extract relative expression values of the genes: indoleamine 2,3‐dioxygenase‐1 (*IDO1*, NM_002164.5), tryptophan 2,3‐dioxygenase‐2 (*TDO2*, NM_005651.1), arylformamidase (*AFMID*, NM_001010982.1), kynurenine‐oxoglutarate transaminase (*KAT*, NM_004059.4), kynureninase (*KYNU*, NM_003937.2), kynurenine 3‐monooxygenase (*KMO*, NM_003679.2), 3‐hydroxyanthranilate 3,4‐dioxygenase (*HAAO*, NM_012205.3), and aminocarboxymuconate‐semialdehyde decarboxylase (*ACMSD*, NM_138326.3). Differential expression analyses in GEO2R were corrected for multiple testing using the Benjamini–Hochberg method. Associations between dichotomized gene expression and clinicopathological variables were evaluated with chi‐square or Fisher's exact tests without additional correction.

Statistical analysis between groups was performed using GEO2R, which applies the Benjamini & Hochberg test and logarithmic transformations when necessary. Genes with adjusted *p*‐values < 0.05 were considered differentially expressed. For correlation analysis, expression values adjusted by the microarray platform were extracted from GEO2R.

Normalized values of relative gene expression were used as numerical variables for correlation analysis among the eight enzymes. In addition, patients were stratified into two groups (low and high expression of the target genes) using a cutoff point determined for each gene based on the median expression value. Correlation analyses were performed to assess the relationship between the relative expression levels of the target genes (low and high) and clinicopathological parameters, including tumor grade, stage, and progression.

### 2.2 Cell Culture

Two human urothelial carcinoma cell lines, RT4 (low‐grade, non‐invasive) and T24 (high‐grade, invasive), were obtained from the American Type Culture Collection (ATCC, Manassas, VA, USA) through the cell bank of the Universidade Federal do Rio de Janeiro, Brazil.

Cell culture was performed in RPMI 1640 medium (Vitrocell, Campinas, Brazil), supplemented with 10% fetal bovine serum (Vitrocell, Campinas, Brazil) and an antibiotic mixture containing penicillin (100 U/mL) and streptomycin (100 μg/mL) (Vitrocell, Campinas, Brazil). The cells were incubated at 37°C in an atmosphere with 5% carbon dioxide.

RT4 or T24 cells were seeded in 6‐well plates at a density of 5 × 10^5^ cells/well. After 24 h of incubation, the culture medium was replaced with medium containing interferon‐gamma (IFN‐gamma, 50 ng/mL, catalog SRP3058, Sigma, St. Louis, USA) and the IDO1 inhibitor, INCB024360 (INCB, 1.0 μM, Cat. 6007, Tocris Bioscience, Bristol, UK). The concentrations of IFN‐gamma and INCB024360 were chosen based on the literature [14, 15]. After 24 h of incubation, the supernatant was collected.

### 2.3 Real Time PCR

Total RNA was extracted from frozen pellets using the PureLink RNA Mini Kit (Thermo Fisher Scientific, Cat. No. 12183018A, Waltham, MA, USA), according to the manufacturer's instructions.

For the reverse transcription step, the High‐Capacity cDNA Reverse Transcription Kit (Thermo Fisher Scientific, Cat. No. 4368814, Waltham, MA, USA) was used, following the manufacturer's instructions.

For real‐time PCR, the SYBR Green kit (Invitrogen, California, USA) was used. Forty cycles of 95°C for 15 s, 60°C for 30 s, and 72°C for 30 s were performed, followed by a melting curve analysis using the QuantStudio 1 equipment (Thermo Fisher Scientific, California, USA). The relative mRNA expression was calculated using the 2^−ΔΔCq method, where ΔΔCq is obtained by normalizing the target gene's Cq values to the endogenous control (TBP housekeeping gene) and comparing the expression to the control group.

The primers used were: *TBP* (NM_003194.4, sense 5′TTCGGAGAGTTCTGGGATTGTA3′, antisense 5′TGGACTGTTCTTCACTCTTGGC3′); *IDO1* (NM_002164.5, sense 5′GGTCATGGAGATGTCCGTAA3′, antisense 5′ACCAATAGAGAGACCAGGAAGAA3′); *AFMID* (NM_001010982.1, sense 5′TTCGACTCCCCCGAATTCCA3′, antisense 5′TTGACACAGGGTCTGCACTG3′), *KAT* (NM_004059.4, sense 5′TAAAGGGACAGGGACTGCTG3′, antisense 5′GGGTTGTAGTCGATCCCGTC3′); *KYNU* (NM_003937.2, sense 5′CACCCTAAGATCTGGCCTGT3′, antisense 5′ATGGAGTGTTTGGCTCCCAG3′); *KMO* (NM_003679.2, sense 5′CTCCGTGTCCTGGGTACAAC3′, antisense 5′GGCCTCCCAGGTTCTTGTAG3′).

### 2.4 Tryptophan Metabolites Measurement by HPLC

Tryptophan metabolites from the kynurenine pathway were analyzed by HPLC (1220 Infinity II LC System, Agilent Technologies) using a standardized protocol. Four detection wavelengths (220, 254, 280, and 314 nm) were evaluated. Standard solutions were prepared with reagents from Sigma‐Aldrich and diluted in RPMI 1640 medium. Samples were deproteinized with trichloroacetic acid, centrifuged, and filtered. The mobile phase consisted of acetonitrile and sodium acetate (4:96 v/v), with separation performed using a Poroshell 120 EC‐C18 column.

### 2.5 Statistical Analysis

Normality was assessed using Kolmogorov–Smirnov and Shapiro–Wilk tests. Spearman's test was used for correlation analysis of non‐normally distributed data. Linear regression and ROC curves were performed. For categorical variables, chi‐square and Fisher's exact tests were applied as needed. Analyses were conducted with IBM SPSS Statistics (Version 22.0).

Dataset analysis was performed in Python on Google Colaboratory (retrieved April 18, 2024), using Pandas and OpenPyxl for data processing, and NumPy and SciPy for statistical analysis. Graphs were generated with Seaborn and Matplotlib.

In vitro studies used Student's *t*‐test or ANOVA with Tukey's or Newman–Keuls post hoc tests, conducted in GraphPad Prism 6.0, which was also used for graph generation.

## 3 Results

### 3.1 Expression of Enzymes Involved in the Trp Catabolism in Human BC (GeoDatasets Analysis)

**FIGURE 2:**
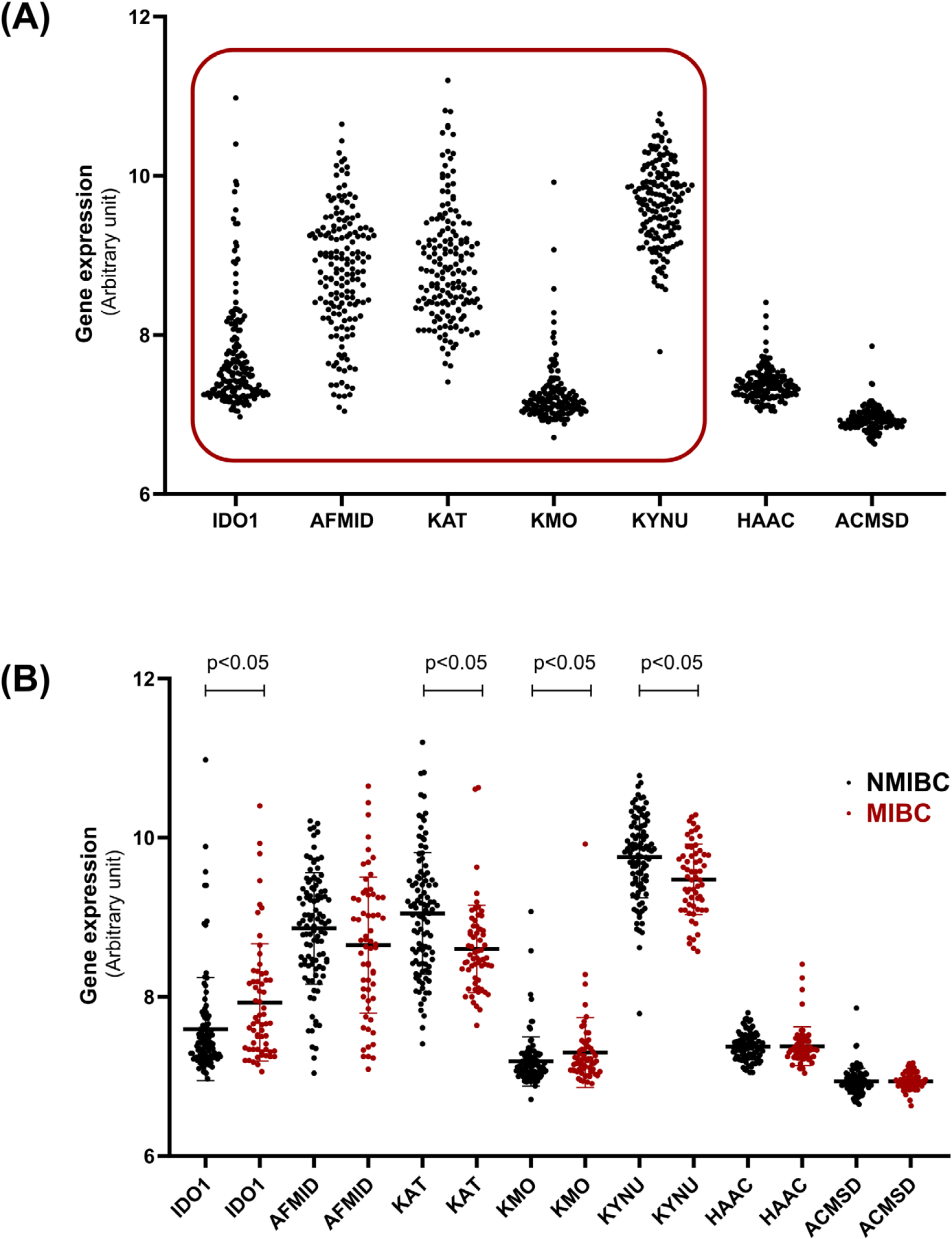
(A) Scatter plot constructed with data from GEO DataSets demonstrating the variance in the expression of enzymes involved in the Kyn pathway (indoleamine 2,3‐dioxygenase‐1 (IDO1), arylformamidase (AFMID), kynurenine–oxoglutarate transaminase (KAT), kynureninase (KYNU), kynurenine 3‐monooxygenase (KMO), 3‐hydroxyanthranilate 3,4‐dioxygenase (HAAO), and aminocarboxymuconate‐semialdehyde decarboxylase (ACMSD)). (B) The same gene expression data, divided into two groups: Non‐muscle‐invasive bladder cancer (NMIBC) and muscle‐invasive bladder cancer (MIBC). A significant difference in the expression of IDO1, KAT, KMO, and KYNU was observed between the two groups.

As previously mentioned, the following results were obtained by exploring the GSE13507 series from GeoDatasets [12, 13]. Figure 2 shows the tumor expression of the seven main enzymes involved in Trp catabolism via the Kyn pathway across all 165 patients with BC. A high variance in the expression of the first four enzymes can be observed.

A correlation analysis revealed that IDO1 expression was positively associated with KMO expression (coefficient 0.23, *p* < 0.05) and negatively with KAT expression (coefficient −0.33, *p* < 0.05). Table 1 shows the chi‐square analysis, where enzyme expression values were categorized into low and high expression. High IDO1 expression was more frequent in MIBC and high‐grade cases, while high KMO expression was more common in MIBC. High expression of KAT and KYNU was more frequently observed in NMIBC and low‐grade cases. No correlation was found between enzyme expression and progression or recurrence.

**TABLE 1:** The chi‐square test was used to analyze the association between the expression of Kyn pathway enzymes and histopathological parameters. The association was considered significant when *p* < 0.05. ns = not significant.

		Stage			Grade			Progression			Recurrence		
	Expression	NMIBC (*n* = 104)	MIBC (*n* = 61)	*p*	Low (*n* = 105)	High (*n* = 60)	*p*	No (*n* = 134)	Yes (*n* = 31)	*p*	No (*n* = 67)	Yes (*n* = 36)	*p*
IDO1	Low	62 (60%)	19 (31%)	< 0.05	63 (60%)	18 (30%)	< 0.05	64 (48%)	17 (55%)	ns	36 (54%)	26 (72%)	ns
	High	42 (40%)	42 (69%)		42 (40%)	42 (70%)		70 (52%)	14 (45%)		31 (46%)	10 (28%)	
TDO2	Low	60 (58%)	22 (36%)	< 0.05	57 (54%)	25 (42%)	ns	71 (53%)	11 (35%)	ns	38 (57%)	21 (58%)	ns
	High	44 (42%)	39 (64%)		48 (46%)	35 (58%)		63 (47%)	20 (65%)		29 (43%)	15 (42%)	
AFMID	Low	48 (46%)	34 (56%)	ns	50 (48%)	32 (53%)	ns	65 (48%)	17 (55%)	ns	30 (45%)	18 (50%)	ns
	High	56 (54%)	27 (44%)		55 (52%)	28 (47%)		69 (52%)	14 (45%)		37 (55%)	18 (50%)	
KAT	Low	40 (39%)	41 (67%)	< 0.05	41 (39%)	40 (67%)	< 0.05	63 (47%)	18 (58%)	ns	23 (34%)	16 (44%)	ns
	High	64 (62%)	20 (33%)		64 (61%)	20 (33%)		71 (53%)	13 (42%)		44 (66%)	20 (56%)	
KMO	Low	58 (56%)	23 (38%)	< 0.05	52 (49%)	29 (48%)	ns	70 (52%)	11 (35%)	ns	38 (57%)	19 (53%)	ns
	High	46 (44%)	38 (62%)		53 (51%)	31 (52%)		64 (48%)	20 (65%)		29 (43%)	17 (47%)	
KYNU	Low	43 (41%)	38 (62%)	< 0.05	43 (41%)	38 (63%)	< 0.05	63 (47%)	18 (58%)	ns	29 (43%)	13 (36%)	ns
	High	61 (59%)	23 (38%)		62 (59%)	22 (37%)		71 (53%)	13 (42%)		38 (57%)	23 (64%)	
HAAC	Low	46 (44%)	36 (59%)	ns	49 (47%)	33 (55%)	ns	60 (45%)	22 (71%)	ns	28 (42%)	17 (47%)	ns
	High	58 (56%)	25 (41%)		56 (53%)	27 (45%)		74 (55%)	09 (29%)		39 (58%)	19 (53%)	
ACMSD	Low	52 (50%)	25 (41%)	ns	49 (47%)	28 (47%)	ns	63 (47%)	14 (45%)	ns	34 (51%)	18 (50%)	ns
	High	52 (50%)	36 (59%)		56 (53%)	32 (53%)		71 (53%)	17 (55%)		33 (49%)	18 (50%)	

**FIGURE 3:**
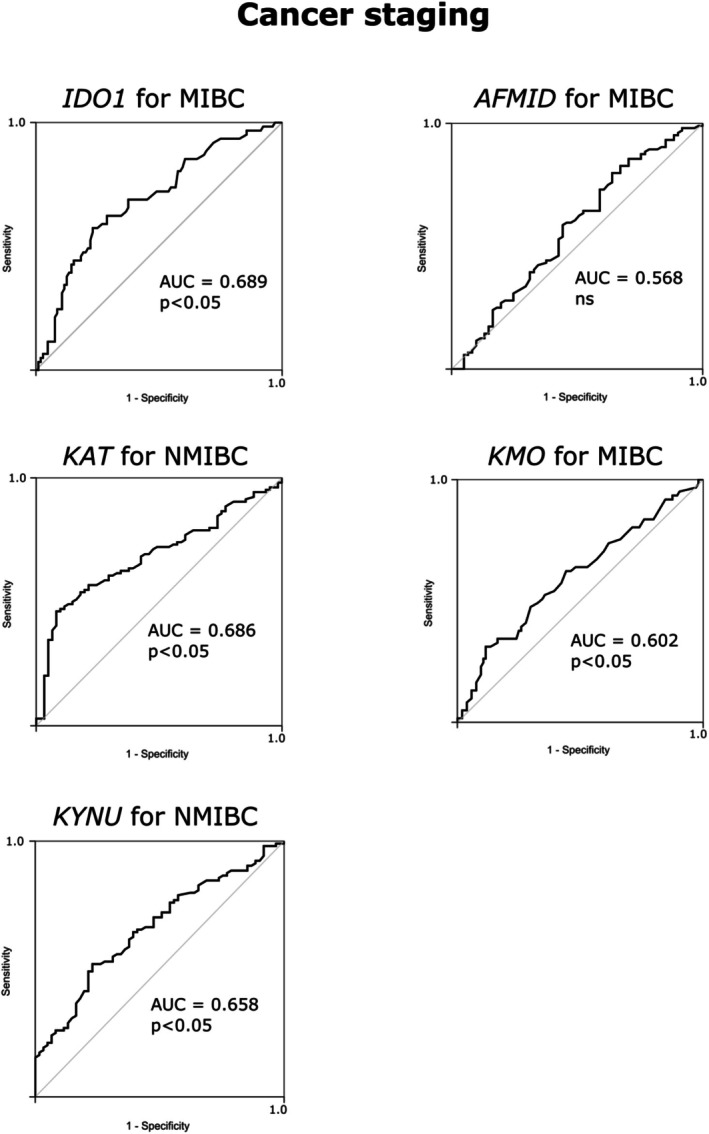
ROC curve demonstrating the evaluation of the expression of enzymes involved in the Kyn pathway for predicting cancer staging. AUC: Area under the curve.

**FIGURE 4:**
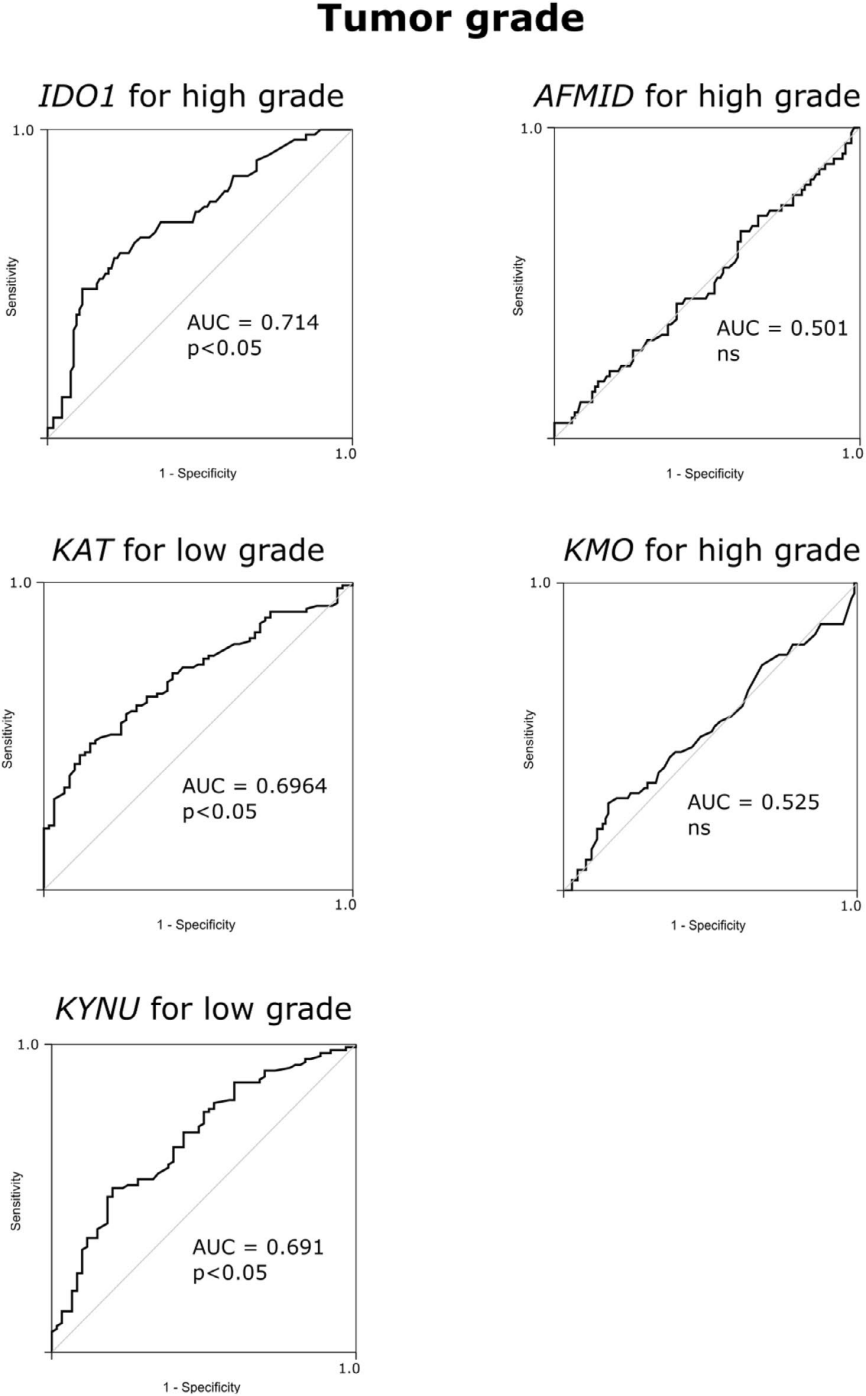
ROC curve demonstrating the evaluation of the expression of enzymes involved in the Kyn pathway for predicting tumor grade. AUC: Area under the curve.

The findings from the Chi‐square analysis were further validated using the ROC curve. The area under the ROC curve (AUC) indicates that IDO1, KAT, KMO, and KYNU have predictive value for tumor stage and grade (Figures 3 and 4).

### 3.2 Expression of Enzymes Involved in the Trp Catabolism in Human Cell Lines of BC

**FIGURE 5:**
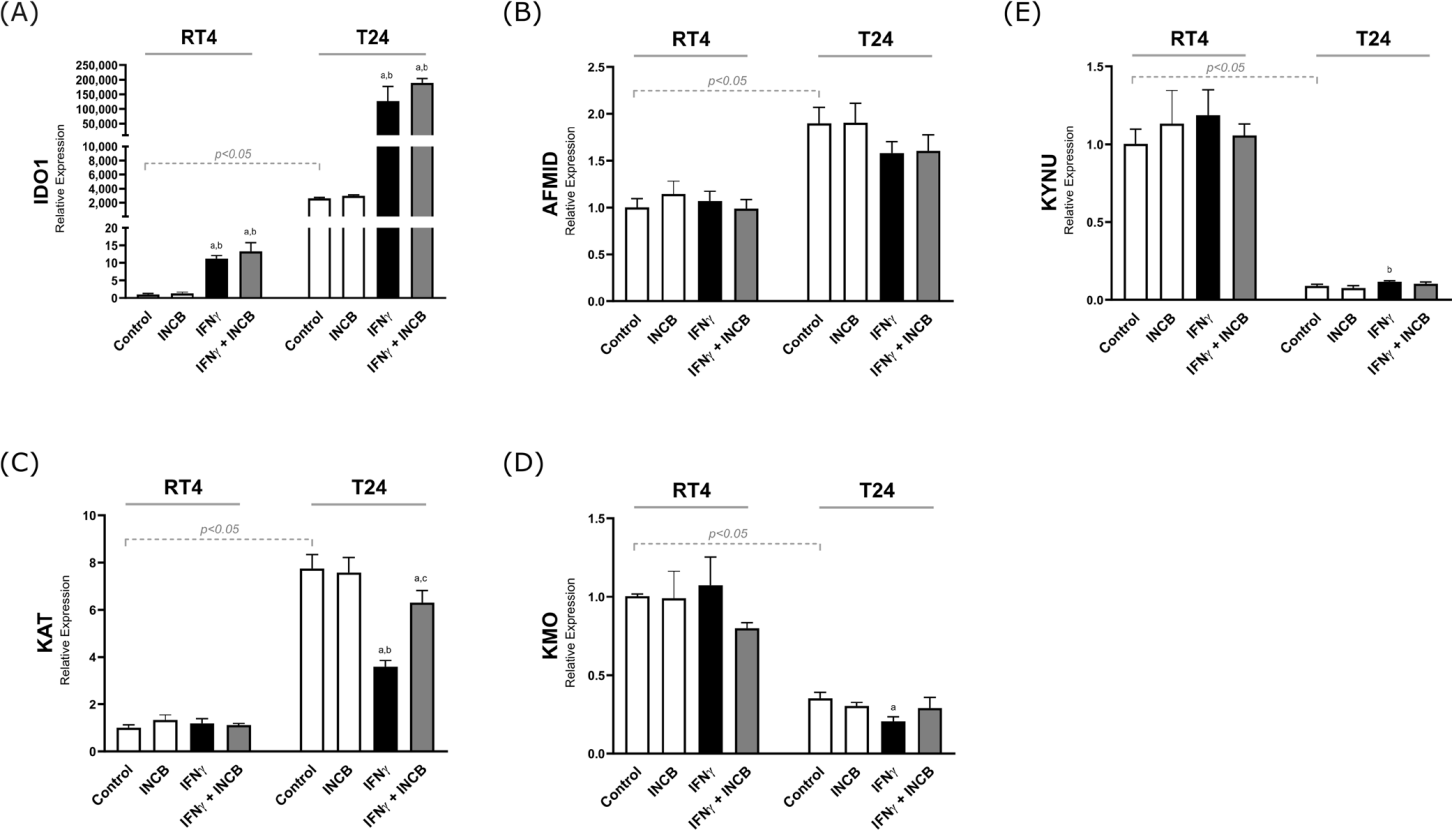
The RT4 and T24 cell lines were incubated with interferon‐gamma (IFN‐γ) and the IDO1 inhibitor INCB024360 (INCB) for 24 h. Total RNA was extracted from frozen cell pellets, reverse transcribed into cDNA, and analyzed via real‐time PCR using SYBR Green. The relative mRNA expression was determined using the 2^−ΔΔCq method, with normalization to the TBP housekeeping gene and comparison to the control group. ^a^ *p* < 0.05 vs. Control; ^b^ *p* < 0.05 vs. INCB; ^c^ *p* < 0.05 vs. IFN‐γ.

The two cell lines used in this study (RT4 and T24) exhibited constitutive expression of *IDO1*, *AFMID*, *KAT*, *KMO*, and *KYNU* (Figure 5). However, when comparing the cell lines in their basal state (Controls), we observed differences in the expression profiles of the five enzymes. RT4 cells showed high expression of *KMO* and *KYNU* and low expression of *IDO1*, *AFMID*, and *KAT* compared to T24 cells.

Additionally, the response to IFN‐gamma varied between the two cell lines. IFN‐gamma significantly increased *IDO1* expression in both lines, but the expression was substantially higher in T24 cells than in RT4 cells (approximately 15 000 times higher in T24). The greater responsiveness of T24 cells to IFN‐gamma was also observed for other enzymes. IFN‐gamma increased *KYNU* expression but significantly decreased *KAT* and *KMO* expression. No effect of IFN‐gamma was noted on the expression of *AFMID*, *KAT*, *KMO*, or *KYNU* in RT4 cells.

The presence of the *IDO1* inhibitor INCB024360 did not affect the response of both cell lines to IFN‐gamma, except for *KAT* expression in T24 cells (Figure 5).

### 3.3 Measurement of TRP Catabolites in the Supernatant of BC Cells

**FIGURE 6:**
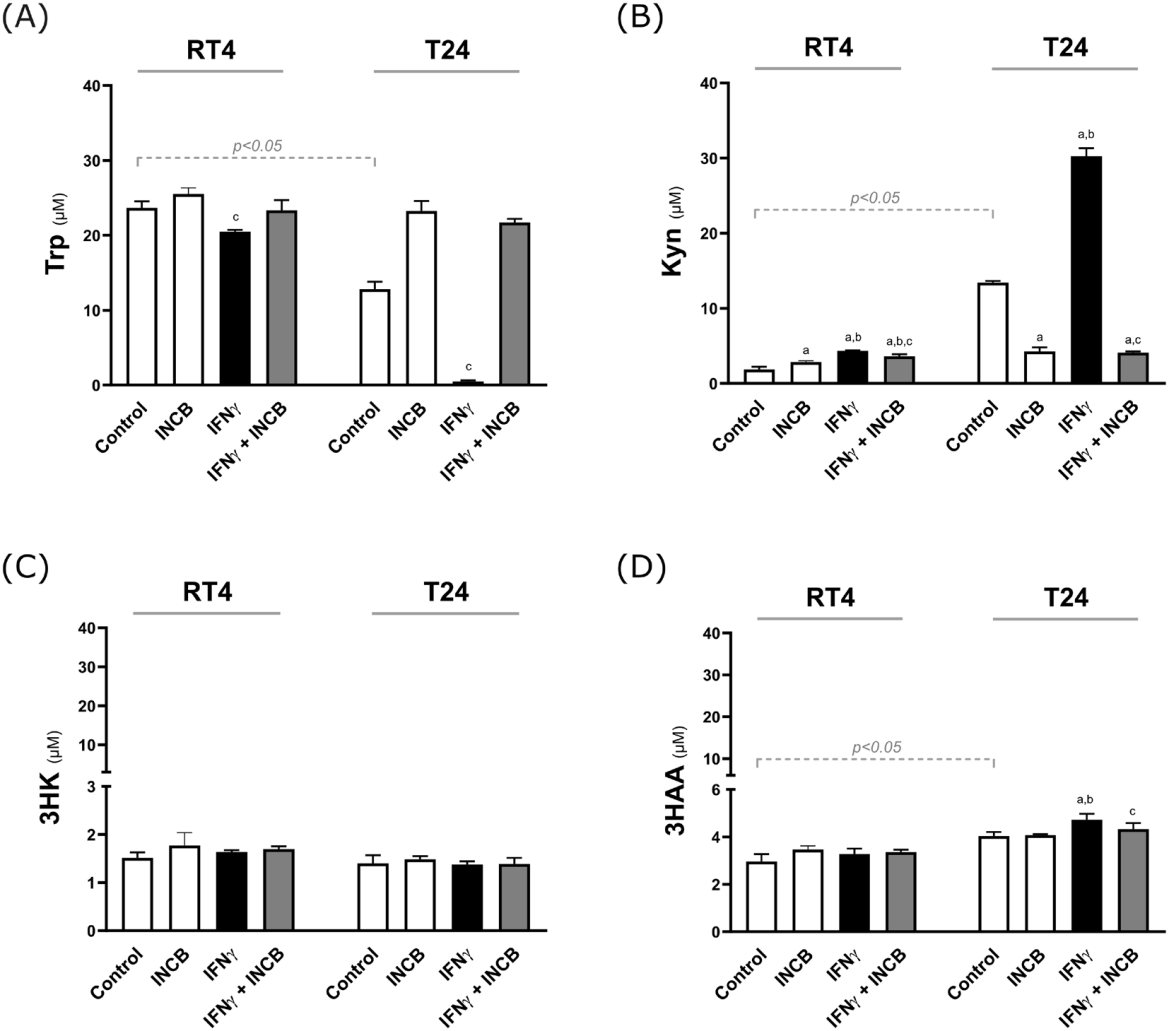
The RT4 and T24 cells were incubated with interferon‐gamma (IFN‐γ) and the IDO1 inhibitor INCB024360 (INCB) for 24 h. Supernatants were collected for metabolite measurement using HPLC: Tryptophan (Trp), L‐kynurenine (Kyn), 3‐hydroxykynurenine (3HK), and 3‐hydroxyanthranilic acid (3HAA). ANOVA was performed between the groups of a specific cell, RT4 or T24. *t*‐test was used to compare the Controls. ^a^ *p* < 0.05 vs. Control; ^b^ *p* < 0.05 vs. INCB; ^c^ *p* < 0.05 vs. IFN‐γ.

Trp and its catabolites were measured in the supernatant of RT4 and T24 cells (Figure 6). Under basal conditions, RT4 cells consumed less Trp than T24 cells, with T24 cells showing a 50% reduction in Trp after 24 h. This decrease was accompanied by higher Kyn production, more pronounced in T24 cells (RT4 Control vs. T24 Control, *p* < 0.05). Incubation with IFN‐gamma reduced Trp in both cell types, with a stronger effect in T24 cells, and increased Kyn production, especially in T24 cells. No effect was observed for 3HK, but 3HAA levels were naturally higher in T24 cells and significantly increased by IFN‐gamma in T24 cells only. The IDO1 inhibitor reversed these effects, confirming that the changes in Trp, Kyn, and 3HAA were due to IDO1 activity.

## 4 Discussion

IDO1 is a key enzyme in Trp catabolism through the Kyn pathway, and its expression has been identified in BC and other neoplasms [16]. IDO1 is the rate‐limiting enzyme in this pathway, generating Kyn derivatives, some of which contribute to its immunomodulatory effects [9]. Our study focused on the downstream pathway following IDO1 activity, aiming to identify differences between tumors at various stages of progression.

Unlike previous studies that examined only IDO1, our work expands the analysis to multiple enzymes across the kynurenine pathway and relates their expression patterns to tumor stage and grade. By integrating transcriptomic data with functional experiments in RT4 and T24 cells, we provide a broader perspective on pathway activity and catabolite production, offering mechanistic insights not previously explored.

### 4.1 Transcriptomic Analysis

The first step was to analyze the expression of enzymes involved in this catabolism in BC cases using the NCBI transcriptomic database, specifically the GSE13507 series, which provides a substantial number of BC cases at various stages along with clinicopathological data [12, 13]. Among the eight enzymes analyzed (Figure 1), the early enzymes in the pathway (IDO1, AFMID, KAT, and KMO) showed high variability in expression across the 165 bc cases. Initially, we could assume that this variation was due to differences in IDO1 expression itself. However, correlation analysis revealed that IDO1 correlates only with KAT (negative) and KMO (positive), suggesting that the factors inducing IDO1 may not directly affect other enzymes or could exert an inhibitory effect, as seen with KAT. Alternatively, IDO1 may regulate the expression of other enzymes via its enzymatic activity.

This variation in enzyme expression has been previously demonstrated. Even in normal human tissues, expression varies, indicating that different tissues generate and utilize Trp catabolites based on differential enzyme expression [10]. In TCGA data, most enzymes are expressed across a range of tumors, with IDO1, TDO2, and AFMID being particularly prominent [10]. In BC, TDO2, AFMID, and KMO were found to be elevated compared to normal bladder tissue [10]. Thus, enzyme expression variability likely determines different Kyn‐derived catabolite combinations, influencing BC pathophysiology.

In our study, we identified enzymes with higher expression in BC and their distribution across tumor stage, grade, progression, and recurrence. High expression of IDO1 and KMO predicted MIBC, while KAT and KYNU predicted NMIBC. Higher IDO1 expression was associated with higher tumor grade, while KAT and KYNU were linked to low grade. IDO1 has already been associated with advanced BC stages [17, 18]. No association with progression or recurrence was found, but this may be due to the limited clinical data available. An important question remains whether the combination of catabolites produced in these tumors could explain the correlation with stage and grade. Measuring catabolites in BC biopsies remains challenging but may become necessary. Because we relied on public databases, we could not correlate enzyme expression with catabolite levels.

### 4.2 Cell Line Findings

Given the challenges of accurately mimicking the tumor microenvironment in culture, we assessed Trp catabolism enzyme expression in BC cell lines. RT4 and T24 are widely used as models for low‐grade (previously grade I) non‐invasive and high‐grade (previously grade III) invasive bladder carcinoma, respectively [19, 20]. The findings from RT4 and T24 cells should be interpreted with caution, as in vitro systems do not replicate the cellular and immunological complexity of the tumor microenvironment. Therefore, immune‐related inferences derived from these cell lines may not fully reflect the behavior of tumors in vivo.

The most surprising result was the difference in basal IDO1 expression between RT4 and T24 cells. While both express IDO1, T24 cells express approximately 2000 times more IDO1 than RT4 cells. Additionally, T24 cells express higher levels of AFMID and KAT, while expressing lower levels of KYNU and KMO. This difference in IDO1 expression has been previously reported [21].

This phenomenon is intriguing, as these cells produce IDO1 without immune‐inflammatory stimuli, despite the fact that its expression is typically induced by IFN‐gamma and other cytokines [22]. One possible explanation could be cytokines in fetal bovine serum used in culture, but our experience suggests that T24 cells express IDO1 even in serum‐free medium.

The regulation of constitutive IDO1 expression in these neoplastic cells remains unclear. Mutations could contribute, as IDO1 expression is influenced by KRas mutations [23]. Alternatively, prior interaction with immune cells could induce and sustain IDO1 expression [24, 25].

Upon IFN‐gamma stimulation, both RT4 and T24 cells increased IDO1 expression, with T24 cells showing a more pronounced response. The presence of INCB did not alter IDO1 expression, likely because it affects enzymatic activity rather than transcription. IFN‐gamma also increased KYNU and decreased KAT and KMO expression in T24 cells, with INCB reversing these effects, showing that IDO1 activity influences subsequent enzyme expression. IFN‐gamma induced increased production of Kyn and 3HAA in T24 cells but had no effect on 3HK levels. These findings suggest that immune system‐driven IDO1 induction promotes specific catabolites that may favor tumor progression.

### 4.3 Implications for Immune Modulation

This could be mediated by the aryl hydrocarbon receptor (AHR), which is implicated in tumorigenesis, progression, and metastasis [26]. AHR activation by Kyn and its derivatives may regulate immune responses, with Kyn promoting immunosuppression in tumors [27, 28]. AHR activation has also been linked to IDO1 induction, suggesting a feedback loop maintaining IDO1 expression. This bidirectional pathway may contribute to sustained IDO1 expression in neoplastic cells [21, 25].

AHR's role in tumor progression and invasiveness has been demonstrated in various cancers, suggesting IDO1's dual role in immune modulation and tumor promotion [29, 30].

### 4.4 Study Limitations

Unfortunately, as this study relied on transcriptomic data, we couldn't determine the predominant catabolites in the tumor microenvironment, which remains a limitation. However, identifying an expression profile with ↑IDO1, ↑KMO, ↓KAT, and ↓KYNU may assist in disease prognosis, alongside other tumor markers. Understanding how the tumor microenvironment influences enzyme expression remains complex, but testing the effects of various cytokines on Kyn pathway enzymes could offer valuable insights.

It is important to note that immune modulation cannot be directly inferred from catabolite patterns alone, as these metabolites may reflect enzymatic regulation, substrate dynamics, and non‐immune pathways. Moreover, because we could not quantify catabolites in tumor tissues, our interpretations should be regarded as hypothesis‐generating rather than definitive.

## Author Contributions

D.E.L.: conceptualization, data curation, analysis, methodology, project administration, original draft. AASS: data curation, methodology, software, project administration, original draft. JLO: data curation, analysis, methodology, software. HR: conceptualization, data curation, analysis, methodology, supervision, review and editing. ACRM: conceptualization, data curation, funding acquisition, investigation, review and editing. CPC: conceptualization, data curation, analysis, validation, review and editing. JPJ: conceptualization, analysis, supervision, validation, original draft, review and editing. HD: conceptualization, analysis, project administration, funding acquisition, investigation, supervision, validation, original draft, review and editing.

## Funding

The study was supported by São Paulo Research Foundation (FAPESP), process number 2022/15575–8, and Conselho Nacional de Desenvolvimento Científico e Tecnológico (CNPq), process number 404044/2021–2.

## Ethics Statement

Ethical committee approval was not required for this study, as it was conducted using a public database and cell culture experiments.

## Conflicts of Interest

The authors declare no conflicts of interest.
